# Initial Accuracy of HIV Rapid Test Kits Stored in Suboptimal Conditions and Validity of Delayed Reading of Oral Fluid Tests

**DOI:** 10.1371/journal.pone.0158107

**Published:** 2016-06-23

**Authors:** Augustine T. Choko, Miriam Taegtmeyer, Peter MacPherson, Derek Cocker, McEwen Khundi, Deus Thindwa, Rodrick S. Sambakunsi, Moses K. Kumwenda, Kondwani Chiumya, Owen Malema, Simon D. Makombe, Emily L. Webb, Elizabeth L. Corbett

**Affiliations:** 1 TB and HIV theme, Malawi Liverpool Wellcome Trust, Blantyre, Malawi; 2 Liverpool School of Tropical Medicine, Liverpool, United Kingdom; 3 Blantyre District Health Office, Ministry of Health, Blantyre, Malawi; 4 Department of HIV, Ministry of Health, Lilongwe, Malawi; 5 London School of Hygiene &Tropical Medicine, London, United Kingdom; 6 College of Medicine, Blantyre, Malawi; 7 Department of Public Health and Policy, University of Liverpool, Liverpool, United Kingdom; 8 Department of Clinical Research, Liverpool School of Tropical Medicine, Liverpool, United Kingdom; Quensland University of Technology, AUSTRALIA

## Abstract

**Objectives:**

To evaluate the effect of storing commonly used rapid diagnostic tests above manufacturer-recommended temperature (at 37°C), and the accuracy of delayed reading of oral fluid kits with relevance to HIV self-testing programmes.

**Design:**

A quality assurance study of OraQuick (OraSure), Determine HIV 1/2^™^ (Alere) and Uni-Gold^™^ (Recombigen^®^).

**Methods:**

Consecutive adults (≥18y) attending Ndirande Health Centre in urban Blantyre, Malawi in January to April 2012 underwent HIV testing with two of each of the three rapid diagnostic test kits stored for 28 days at either 18°C (optimally-stored) or at 37°C (pre-incubated). Used OraQuick test kits were stored in a laboratory for delayed day 1 and subsequent monthly re-reading was undertaken for one year.

**Results:**

Of 378 individuals who underwent parallel testing, 5 (1.3%) were dropped from the final analysis due to discordant or missing reference standard results (optimally-stored Determine and Uni-Gold). Compared to the diagnostic reference standard, OraQuick had a sensitivity of 97.2% (95% CI: 93.6–99.6). There were 7 false negative results among all test kits stored at 37°C and three false negatives among optimally stored kits. Excellent agreement between pre-incubated tests and optimally-stored tests with Kappa values of 1.00 for Determine and Uni-Gold; and 0.97 (95% CI: 0.95; 1.00) for OraQuick were observed. There was high visual stability on re-reading of OraQuick, with only 1/375 pre-incubated and 1/371 optimally-stored OraQuick kits changing from the initial result over 12 months.

**Conclusion:**

Erroneous results observed during HIV testing in low income settings are likely to be due to factors other than suboptimal storage conditions. Re-reading returned OraQuick kits may offer a convenient and accurate quality assurance approach, including in HIV self-testing programmes.

## Introduction

Globally, an estimated 45% of people living with HIV are aware of their HIV status[1], far below the 90% target set out recently by WHO and UNAIDS[1]. The greatest numbers of people living with undiagnosed HIV are in sub-Saharan Africa [1], where fragile health and laboratory systems have hindered efforts to scale up HIV testing coverage [2]. In low-income, high HIV burden countries with limited technological infrastructure, rapid diagnostic tests (RDTs) are widely used in diagnostic algorithms [1].

RDT accuracy may be undermined by poor operator practice [3, 4], and by storage at ambient temperatures above manufacturer guidelines [5]. Poor accuracy of RDTs and subsequent misclassification can result in a) if falsely diagnosed HIV-positive, unnecessary worry and initiation on antiretroviral therapy; or b) if falsely diagnosed HIV-negative, missed opportunities for linkage to HIV care and prevention services[6, 7].

The scale-up of HIV self-testing (HIVST) using oral fluid testing will pose additional challenges to maintaining accuracy in HIV testing programmes. Self-testing is defined by an individual performing and interpreting their own HIV test result [8]. Oral fluid RDTs intended for self-testing may be stored in uncontrolled settings (e.g. people’s houses) for prolonged periods before use and are less amenable to formal quality assurance (QA) programmes [9]. We therefore set out to evaluate two aspects of RDT kit stability: the effect of prolonged field exposure to high temperature on the accuracy of whole blood and oral fluid RDTs; and the stability of oral RDT results with delayed visual re-reading.

## Methods

### Study design and participants

A quality assurance study of three HIV RDTs (OraQuick: OraSure Technologies, USA; Determine 1/2^™^: Alere, Waltham, USA; and Uni-Gold^™^: Recombigen^®^ HIV, Trinity Biotech, Bray, Ireland) was conducted at Ndirande Health Centre in Blantyre, Malawi. Participants were recruited between 14^th^ January and 5^th^ March 2012 and visual re-reading of OraQuick kits continued until 30^th^ April 2013. Participants were recruited from both HIV testing and counselling (HTC) and antiretroviral treatment (ART) clinics. All consecutive adults (≥18 years) presenting for HIV testing or ART care during the study period were invited to participate regardless of previous history of HIV testing.

### Definitions

Three HIV rapid diagnostic tests were evaluated in this study: OraQuick ADVANCE HIV I/II^™^ (OraSure Technologies, Bethlehem, USA; assembled in Thailand), Determine 1/2^™^ (Alere, Waltham, USA) and Uni-Gold^™^ Recombigen^®^ HIV, Trinity Biotech, Bray, Ireland).

#### Optimally-stored index tests

Intact packages of rapid diagnostic tests stored on-site in a laboratory storage facility at a temperature of 18°C as recommended by the manufacturer (2–27°C, 2–27°C and 2–30°C for OraQuick, Uni-Gold and Determine, respectively).

#### Pre-incubated index tests

Intact packages of rapid diagnostic tests pre-incubated for 28 days at 37°C in an incubator in a laboratory to mimic higher than manufacturer-specified conditions.

### Index tests and reference standard

Individuals who gave written or witnessed (thumb print) consent underwent HIV testing with six RDTs: two each of OraQuick, Determine 1/2^™^ and Uni-Gold^™^. Pairs of RDTs had been either optimally-stored or pre-incubated prior to use. One study nurse who had been trained in the use of RDTs performed each test in parallel on the same participant using a venous blood sample (Determine and Uni-Gold) and oral fluid (OraQuick).

The reference standard for HIV diagnosis comprised of a parallel HIV testing algorithm consisting of two simultaneous HIV RDTs used in the Malawi National algorithm (Determine and Uni-Gold^™^). Both RDTs included in the reference standard were required to return similar results or a repeat of all optimally-stored tests was undertaken to resolve discordance.

### Procedures

A study nurse sampled blood using venipuncture for use with blood RDTs and asked the participant to perform an oral swab for use with OraQuick. The nurse read all six test kits at the recommended time-points and gave results to the participant based on the reference standard. Results were recorded as reactive or non-reactive, with a repeat in case of an invalid result. Weakly reactive bands were recorded as positive. All blood RDTs and any remaining blood samples collected were disposed of by the nurse at the study recruitment site.

Oral test kits (OraQuick), both optimally stored and pre-incubated at high temperatures, were transported to a research laboratory for subsequent re-reading in order to assess visual stability. The oral test kits were read upon arrival by a laboratory technician blinded to the original results from the nurse. Test kits were then re-read at one week, two weeks, one month, 3 months, 6 months and 12 months to assess visual stability, with the reader blinded to previous read results.

### Ethical considerations

Ethical approval was obtained from the University of Malawi, College of Medicine Research and Ethics Committee (COMREC) under ethical approval number: P.03/12/1186. All participants gave written or witnessed (thumb print) consent.

### Statistical methods and sample size considerations

Stata version 13.0 (Stata Corp, Texas, USA) was used for data analysis. Estimates of sensitivity and specificity with 95% confidence intervals (CI) for each index test and storage/read condition were computed by comparing pre-incubated kits and optimally stored OraQuick kits against the reference diagnostic algorithm comprising of Determine and Uni-Gold in parallel. Further analysis of reliability was conducted by computing a Kappa statistic by comparing pre-incubated and optimally-stored RDTs.

A total of 200 HIV positive and 200 HIV negative participants provided precision of +/- 2% around our assumed estimate of sensitivity of 98% (95% binomial exact CI: 94%-99%) and specificity of 99% for each RDT comparison with the reference standard.

## Results

### Characteristics of study participants

**Fig 1:**
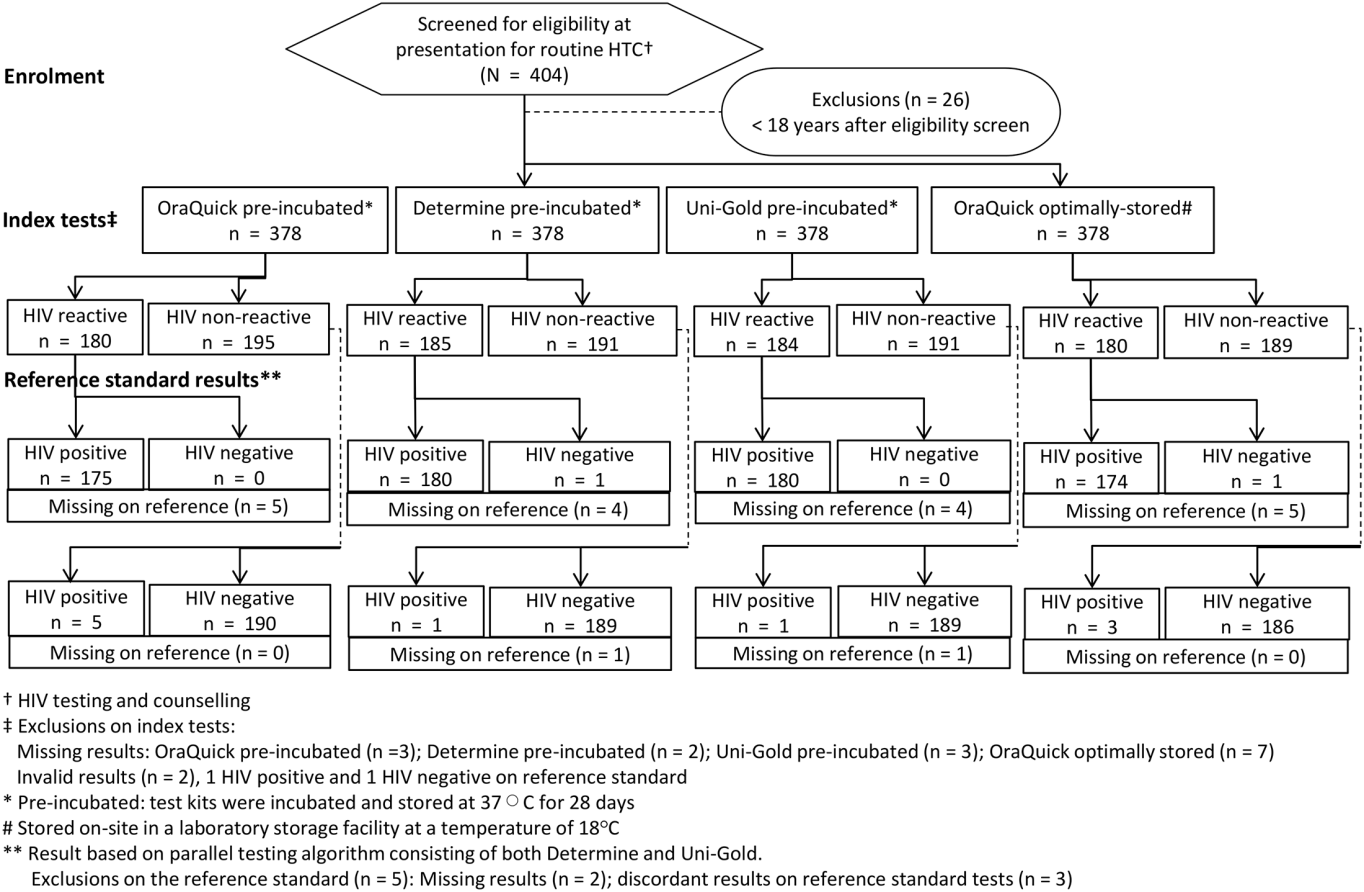
Flow of participant recruitment and HIV testing.

Of 404 individuals screened for eligibility, a total of 378 (93.6%) were recruited into the study (Fig 1). Median age was 31 years (IQR 25–40), and 198 (52.4%) participants were male. The majority (242, 64.0%) of participants had previously tested for HIV, with comparable proportions of prior testing among men (121/238, 50.8%) and women (117/238, 49.2%; p = 0.096). A total of 108 (28.6%) HIV-positive individuals were already on ART.

### Sensitivity and specificity of index tests

Results from 5/378 (1.3%) participants were excluded from the computation of sensitivity and specificity (3 excluded were due to discordant results between Determine and Uni-Gold and 2 due to missing result from ≥ 1 reference standard test). All RDTs had a sensitivity > 98.0% except pre-incubated OraQuick with sensitivity 97.2% (95% CI: 93.6–99.6) Table 1. Specificity was higher than 99.5% for all RDTs. There were seven false negative results in total across all the pre-incubated RDTs compared with three false negatives when RDTs were optimally stored.

**Table 1:** Sensitivity and specificity compared to a reference standard (parallel testing).

RDT Kit	TP/FN	Sensitivity (95% CI)	TN/FP	Specificity (95% CI)
**OraQuick Pre-incubated**	175/5	97.2 (93.6–99.6)	190/0	100 (98.1–100)
**Determine** Pre-incubated	180/1	99.4 (97.1–100)	189/1	99.5 (97.1–100)
**Uni-Gold** Pre-incubated	180/1	99.4 (97.0–100)	189/0	100 (98.1–100)
**OraQuick** Optimally-stored	174/3	98.3 (95.2–99.7)	186/1	99.5 (97.1–100)

RDT: rapid diagnostic test

CI: confidence interval; TP: true positive; FN: false negative; TN: true negative; FP: false positive

### Reliability of pre-incubated RDTs

There was excellent agreement between pre-incubated and optimally-stored RDTs with Kappa of 1.00 for Determine and Uni-Gold (Table 2). Similar results were observed for pre-incubated OraQuick, with Kappa of 0.97 (95% CI: 0.95; 1.00).

**Table 2:** Reliability comparison of optimally stored and pre-incubated tests.

RDT Kit	Both positive	Both negative	Positive: negative	Negative: positive	Kappa (95% CI)
**Determine**	185	191	0	0	1.00 (1.00; 1.00)
**Uni-Gold**	184	191	0	0	1.00 (1.00; 1.00)
**OraQuick**	176	187	4	1	0.97 (0.95; 1.00)

RDT: rapid diagnostic test

CI: confidence interval

### Visual stability of OraQuick RDT over12 months

In total, 375/378 pre-incubated OraQuick and 371/378 optimally-stored OraQuick RDTs were re-read over a period of 12 months to assess the stability of visual reads. There was very high reproducibility of results, with only 1/375 pre-incubated and 1/371 optimally-stored OraQuick kits changing from the initial result. Both kits were initially read as positive and had at least one subsequent positive read following the false negative read, which was recorded within a month of testing.

## Discussion

The main findings of this study were that the contribution of suboptimal storage to misclassification of HIV results is likely to be relatively minor, and that re-reading of used oral fluid tests (OFT) may be a feasible and valid option for implementing quality assurance (QA) of OraQuick test kits as these are scaled up for use by less qualified personnel, including during HIV self-testing [9].

Pre-incubating HIV RDTs at 37°C for 28 days did not substantially affect their diagnostic accuracy, and, in all but two cases (both known to be taking ART), initial visual read results on OraQuick test kits were stable over the one-year follow up period in this study. The results from our study demonstrate the stability of OraQuick, Determine and Uni-Gold when pre-incubated at 37°C for 28 days, which is an important consideration in resource poor settings where poor storage conditions are common [5].

The scale up of HTC in Africa was not initially accompanied by systematic QA or retraining, but the need to build systematic proficiency testing into routine HIV testing programmes has become strikingly apparent with reports of poor operator practice and high rates of misclassification in many African national programmes [10, 11]. Lessons from this experience need to be applied to HIVST and other types of highly decentralised community-based HIV testing services.

Our results, showing robustness of the kits to poor storage and late read, are consistent with current understanding that identifies poor operator practice as the major determinant of misclassification [10, 11], in part reflecting “task-shifting” of HTC to cadres such as lay counsellors. The introduction of very simple oral fluid test kits will enable further task-shifting including as part of HIV self-testing (HIVST) [9]. Systems for providing external QA for oral fluid tests and HVST are not yet well established. These data provide reassurance that countries currently revising QA systems to accommodate oral fluid tests and HIVST could consider including systems based on visual re-reading. While outside of manufacturer’s recommendations, our results suggest that for OraQuick at least, this provides a simple and practical approach to monitoring operator-dependant misclassifications if used kits can be retrieved along with the users’ interpretation of results. In this context, we have previously reported a high willingness to return HIVST kits to the distributor, together with a self-completed questionnaire that includes results interpretation [9].

More work is needed to develop fully comprehensive external quality assurance (EQA) systems for oral fluid RDTs. False-positive results have been described for OFTs, particularly in the context of pregnancy and concomitant infections [12, 13]. With respect to sensitivity, subsets where oral-based RDTs should be used with caution because of reports of lower than expected sensitivity [14] are patients at high risk of acute HIV infection [15] and those established on ART. In this study, we report one potential false-positive from 369 optimally and 375 sub-optimally stored OFT kits, and a suggestion that poor storage conditions may reduce sensitivity but with no obvious effects on specificity. We also report two participants already on ART who had an observed change in the result of their oral test kits when re-read over time. Otherwise, there was minimal change in the interpretation of used OraQuick RDT results when read up to one year after testing in this relatively small sample.

The implementation of a multistep approach to HIV testing has been recommended [1], and it will be vital to have rigorous QA mechanisms in place in an effort to reduce error amongst testers in both home-based and laboratory settings. Re-evaluation of used test kits could contribute to internal and external quality assurance programs [1, 16].

There were three notable limitations in this study. First of all, our reference standard was not carried out separately from the index test rendering blinding of index tests impossible. Secondly, we did not exclude patients already on ART, despite the known issues of low sensitivity of RDTs in this important subgroup. Lastly, we did not measure the impact of humidity a long with temperature in this study.

In summary, this study has shown that prolonged exposure of RDTs to temperatures above manufacturer’s recommendation did not substantially affect the sensitivity and specificity of commonly used RDTs including OraQuick OFT. Furthermore, visual interpretation of OraQuick results was highly stable for 12 months after testing, supporting the use of re-reading of used test kits as part of programmatic quality assurance.
